# Impact of Vaptans on Clinical Outcomes in Cirrhosis Patients: A Meta-Analysis of Randomized Controlled Trials

**DOI:** 10.3389/fphar.2019.01365

**Published:** 2019-11-20

**Authors:** Miao Li, Zhuofang Bi, Zicheng Huang

**Affiliations:** ^1^Department of Gastroenterology, the Sixth Affiliated Hospital, Sun Yat-sen University, Guangzhou, China; ^2^Guangdong Provincial Key Laboratory of Colorectal and Pelvic Floor Diseases, the Sixth Affiliated Hospital, Sun Yat-sen University, Guangzhou, China; ^3^Department of Ultrasonography, the Sihui People’s Hospital, Zhaoqing, China

**Keywords:** cirrhosis, ascites, V2-receptor antagonist, mortality, meta-analysis

## Abstract

**Background:** Vaptans have been confirmed to mobilize ascites and improve hyponatremia in cirrhosis patients. However, the effects of vaptans on all-cause mortality, ascites-related complications, and adverse events in cirrhosis patients have not been fully determined.

**Objectives:** To systematically evaluate the impact of vaptans on the clinical outcomes in patients with cirrhosis.

**Materials and Methods:** A systematic review and meta-analysis was performed. The PubMed, Embase, and Cochrane’s Library electronic databases were systematically searched for randomized controlled trials (RCTs) investigating the clinical efficacy of vaptans in cirrhosis patients. The results were pooled with a random-effect model.

**Results:** Eighteen RCTs containing 3,059 cirrhosis patients with ascites and/or hyponatremia were included. Meta-analysis showed that vaptans did not significantly affect the risk of all-cause mortality (RR: 1.02, 95% CI: 0.87 to 1.08, p = 0.83; I^2^ = 2%), consistent with studies with short-term (< 26 weeks) and long-term (≥ 26 weeks) follow-up durations. Additionally, vaptans did not affect the incidence of variceal bleeding (RR: 0.96, p = 0.86), showed a trend of reduced incidence of hepatic encephalopathy (RR: 0.86, p = 0.09), significantly reduced the incidence of spontaneous bacterial peritonitis (RR: 0.75, p = 0.03), but did not significantly affect the risk of hepatorenal syndrome or renal failure (RR: 1.09, p = 0.36). Vaptans did not affect the incidence of adverse events in cirrhosis patients.

**Discussion:** Treatment with vaptans is not associated with improved survival in cirrhosis patients, although it may reduce the risk of hepatic encephalopathy and spontaneous bacterial peritonitis in these patients. The limitations of the current study include limited number of available studies, small sample sizes of the included studies, variations of baseline patient characteristics, and differences in the dose and duration of vaptans.

## Introduction

Cirrhosis patients have a greater risk of developing ascites (Fortune and Cardenas, 2017). In fact, it has been confirmed that up to 60% of cirrhosis patients will develop ascites within 10 years of diagnosis (Neong et al., 2019). Ascites are associated with increased risk of various serious complications, which may later contribute to a significantly increased mortality in these patients (Ennaifer et al., 2016; Neong et al., 2019). Medical treatments for ascites in cirrhosis patients mainly consist of diuretics (Annamalai et al., 2016; Zhao et al., 2018). However, the efficacy of conventional diuretics for ascites is limited and patients receiving these medications are vulnerable to adverse effects such as electrolyte imbalance, renal dysfunction, and hypotension (Annamalai et al., 2016; Zhao et al., 2018). On the other hand, a considerable number of patients with cirrhosis and ascites that receive conventional diuretics will eventually respond poorly to these medications, probably due to hyponatremia caused by conventional diuretics (Van Blijderveen et al., 2014). Hyponatremia in cirrhosis patients with ascites leads to deteriorated water retention and has been confirmed to be an independent risk factor of mortality (Serste et al., 2012; Sinha and Ko, 2015).

Vaptans are a category of nonpeptide vasopressin receptor antagonists (Veeraveedu et al., 2010). By blocking the V2 receptors in the renal collecting ducts, vaptans increase free water clearance from the kidneys (Decaux et al., 2008). Through this hypotonic diuretic effect, vaptans have been confirmed to mobilize ascites and improve hyponatremia in cirrhosis patients (Dahl et al., 2012; Yan et al., 2015). Since it has been demonstrated that both ascites and hyponatremia are significant risk factors of mortality in cirrhosis patients, vaptans are expected to improve the prognosis for these patients. However, the use of vaptans has also been associated with some adverse events such as osmotic demyelination, myelinolysis, and liver injuries (Muto et al., 2017), and a previous meta-analysis showed that treatment with vaptans increased the risk of total adverse events in cirrhosis patients (Dahl et al., 2012). In addition, RCTs evaluating the efficacy of vaptans in cirrhosis patients reported inconsistent results (Gerbes et al., 2003; Wong et al., 2003; Gines et al., 2008a; Gines et al., 2008b; Wong et al., 2009; Gines et al., 2010; Wong et al., 2010; Cardenas et al., 2012; Wong et al., 2012; Okita et al., 2014; Sakaida et al., 2014; Rai et al., 2017; Uojima et al., 2017; Wang et al., 2018a; Wang et al., 2018b; Wang et al., 2019a) and the overall effect of vaptans on clinical outcomes in cirrhosis patients remains to be determined. Two previous meta-analyses including 12 and 14 RCTs have been published evaluating the efficacy of vaptans for cirrhosis patients (Dahl et al., 2012; Yan et al., 2015). Both concluded that although vaptans have beneficial effects on ascites and hyponatremia, the use of vaptans is not associated with reduced overall mortality or complications of ascites in these patients (Dahl et al., 2012; Yan et al., 2015). However, a few RCTs have been published since then (Rai et al., 2017; Uojima et al., 2017; Wang et al., 2018a; Wang et al., 2018b; Wang et al., 2019a), and in view of the limited scale of the previous RCTs, including these new studies may yield a more reliable result.

Therefore, the objective of this updated systematic review and meta-analysis was to evaluate the impact of vaptans on the clinical outcomes in patients with cirrhosis.

## Methods

This meta-analysis was designed, performed, and presented in accordance with the Preferred Reporting Items for Systematic Reviews and Meta-Analyses (PRISMA) statement (Moher et al., 2009) and Cochrane’s Handbook guidelines (Higgins and Green, 2011). No registration was made for the protocol of the meta-analysis. We did not perform trial registry search because no missing information seems to be retrieved from registries.

### Database Search

We performed the initial electrical database search of PubMed, Embase, and Cochrane’s Library *via* a combination of the following terms: 1) “vaptan” OR “tolvaptan” OR “satavaptan” OR “lixivaptan” OR “conivaptan” OR “mozavaptan” OR “vasopressin V2 receptor antagonist” OR “OPC 31260” OR “VPA 985” OR “RMJ-351647” OR “nonpeptide arginine vasopressin antagonist”; 2) “cirrhosis” OR “cirrhotic” OR “liver fibrosis” OR “ascites” OR “hepatic edema” OR “hyponatremia” OR “liver fibrosis” OR “hepatic edema” OR “hyponatremia”; and 3) “randomized” OR “randomly” OR “randomized” OR “random.” The search was limited to clinical studies in humans published in English or Chinese. The final literature search was performed on June 25th, 2019. We did not contact the authors of retrieved studies to identify additional studies.

### Inclusion and Exclusion Criteria

Studies were included if they met the following criteria: 1) designed as a parallel group RCT; 2) included adult patients with hepatic cirrhosis; 3) included an intervention group treated with vaptans or a control placebo group, standard medical treatment, or conventional diuretics; 4) involved treatment and observational duration lasting for at least one week; and 5) reported at least one of the following clinical outcomes: all-cause mortality, ascites related complications including variceal bleeding, hepatic encephalopathy, spontaneous bacterial peritonitis, hepatorenal syndrome or renal failure, or adverse events. Reviews, crossover trials, preclinical studies in animals, studies with a single dose of vaptans, and repeated reports of already included RCTs were excluded.

### Study Outcomes, Data Extraction, and Quality Evaluation

The primary outcome of the study was the impact of vaptans on all-cause mortality in cirrhosis patients. The secondary outcomes included the incidence of ascites-related complications including variceal bleeding (VB), hepatic encephalopathy (HE), spontaneous bacterial peritonitis (SBP), hepatorenal syndrome, and renal failure (RF). The safety outcomes included the incidence of any adverse events or serious adverse events. The diagnostic criteria for ascites-related complications were consistent with those applied in the original studies. The definitions of serious adverse events were also consistent with those applied in the original studies, and often consisted of adverse events that required discontinuation of the medications. We extracted the following study characteristics for each RCT: 1) first author and publication year; 2) design characteristics: single-blind, double-blind, or open-label; 3) patient characteristics: number, age, gender, and proportion of patients with ascites or hyponatremia; 4) intervention characteristics: name, dose, and treatment duration of vaptans, and control regimens; and 5) follow-up durations. The quality of the included RCTs was evaluated using the Cochrane’s risk of bias tool (Higgins and Green, 2011), which is based on the following seven domains: random sequence generation, allocation concealment, blinding in performance, blinding in outcome detection, incomplete outcome data, reporting bias, and the potential risk of other biases. The processes of database search, study identification, data extraction, and quality evaluation were independently performed by two authors. Discussion with a third author was indicated when discrepancies occurred.

### Statistical Analyses

The statistical analyses were performed with RevMan software (Version 5.1; Cochrane Collaboration, Oxford, UK) and Stata software (Version 12.0; Stata Corporation, College Station, TX). The categorized variables were analyzed using the risk ratio (RR) and 95% CI. Heterogeneity among the included RCTs was evaluated with Cochrane’s Q test (Higgins and Green, 2011), and a P < 0.10 indicated significant heterogeneity. We also used the I^2^ statistic, which describes the percentage of total variation across studies that is due to heterogeneity rather than chance (Higgins et al., 2003), as an indicator of heterogeneity. An I^2^ > 50% indicated significant heterogeneity. A random-effect model was applied to pool the results since this model was expected to incorporate potential heterogeneity and to result in a generalized outcome (Ma et al., 2018). Predefined subgroup analyses were performed to evaluate the influence of the follow-up duration on the mortality outcome (Moher et al., 1998). Sensitivity analyses were performed to evaluate the impact of vaptans on the mortality outcome in cirrhosis patients with hyponatremia before treatment. The potential publication bias for the meta-analysis of each outcome was evaluated through visual inspection of the symmetry of the funnel plots, as well as Egger’s regression test (Egger et al., 1997). A P value < 0.05 indicated statistical significance.

## Results

### Database Search Results

**Figure 1:**
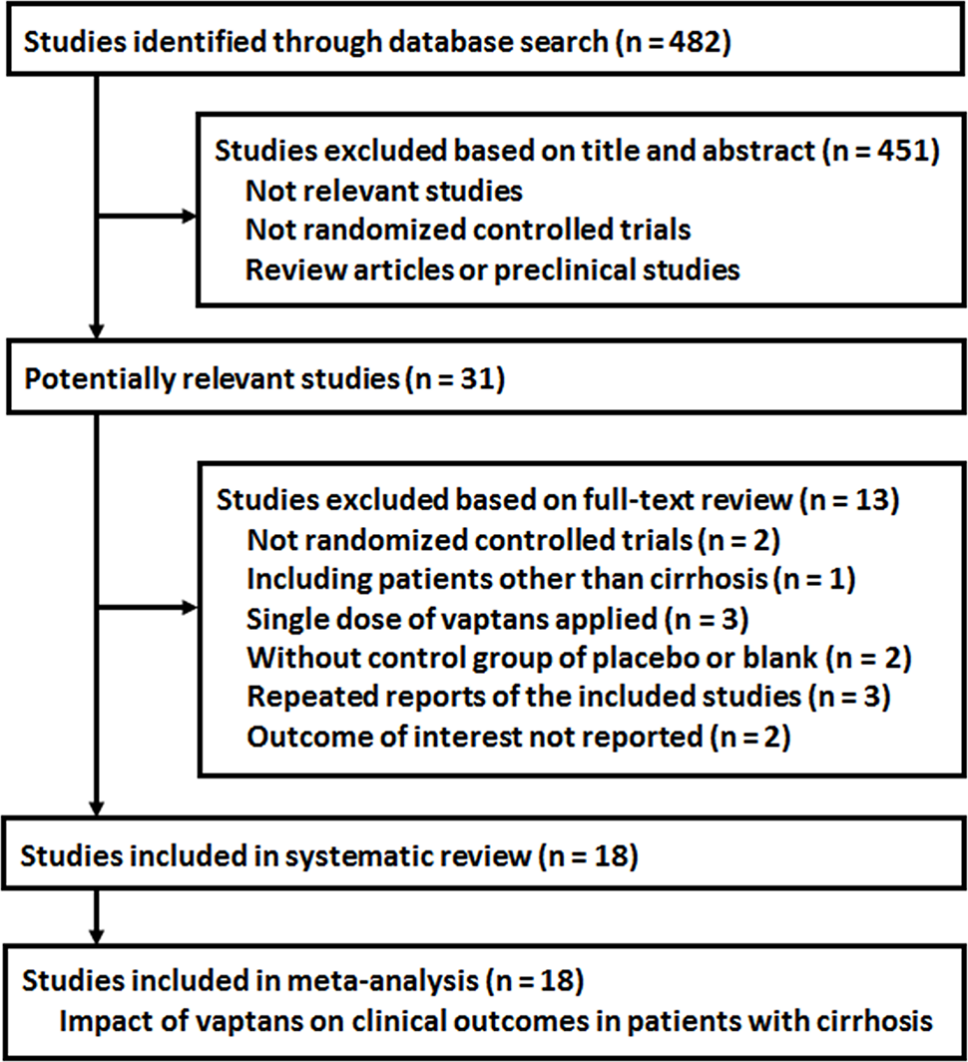
Process of database search and study identification.

The process of database search is summarized in **Figure 1**. Briefly, 482 RCTs were obtained in the initial database search, and 451 were further excluded based on analyses of the titles and abstracts, mostly because these studies were not relevant to the current study objective. Of the remaining 31 studies that underwent full-text review, 13 studies were excluded because they were not RCTs (n = 2), included patients with diseases other than cirrhosis (n = 1), included only a single dosage of vaptans (n = 3), used controls other than placebo or standard treatments (n = 2), repeated reports of the included RCTs (n = 3), or lacked available outcome data (n = 2). Since one article reported three independent studies (Wong et al., 2012), a total of 18 RCTs from 16 publications were included in the analysis (Gerbes et al., 2003; Wong et al., 2003; Gines et al., 2008a; Gines et al., 2008b; Wong et al., 2009; Gines et al., 2010; Wong et al., 2010; Cardenas et al., 2012; Wong et al., 2012; Okita et al., 2014; Sakaida et al., 2014; Rai et al., 2017; Uojima et al., 2017; Wang et al., 2018a; Wang et al., 2018b; Wang et al., 2019a).

### Study Characteristics and Quality Evaluation

The characteristics of the included RCTs are presented in **Table 1**. Overall, 18 multi-center RCTs, including 3,059 cirrhosis patients with ascites and/or hyponatremia were included. The mean ages of the patients for each study varied from 43 to 70 years, and the proportion of male patients varied from 56 to 80%. The baseline serum sodium varied between 124 and 137 mmol/L. Lixivaptan was used in two RCTs (Gerbes et al., 2003; Wong et al., 2003), satavaptan in eight RCTs (Gines et al., 2008a; Gines et al., 2008b; Wong et al., 2009; Gines et al., 2010; Wong et al., 2010; Wong et al., 2012), and tolvaptan in the remaining eight RCTs (Cardenas et al., 2012; Okita et al., 2014; Sakaida et al., 2014; Rai et al., 2017; Uojima et al., 2017; Wang et al., 2018a; Wang et al., 2018b; Wang et al., 2019a). The treatment duration varied from 1 to 52 weeks. The details of the study quality evaluation are presented in **Table 2**. Briefly, 12 studies were double-blinded RCTs (Gerbes et al., 2003; Gines et al., 2008b; Gines et al., 2010; Wong et al., 2010; Cardenas et al., 2012; Wong et al., 2012; Okita et al., 2014; Sakaida et al., 2014; Wang et al., 2018a; Wang et al., 2018b), three were single-blinded (Wong et al., 2003; Gines et al., 2008a; Wong et al., 2009), and another three were open-label (Rai et al., 2017; Uojima et al., 2017; Wang et al., 2019a). Six studies reported the methods used for random sequence generation (Gerbes et al., 2003; Gines et al., 2008b; Gines et al., 2010; Wong et al., 2010; Rai et al., 2017; Uojima et al., 2017) and three studies (Wong et al., 2010; Rai et al., 2017; Uojima et al., 2017) reported the details of allocation concealment.

**Table 1:** Characteristics of the included randomized control trials.

Study	Design	Patients number	Ascites	Hyponatremia	Mean age	Male	Baseline serum sodium	Intervention	Control	Treatment duration	Follow-up duration
			%		Years	%	mmol/L			Weeks	Weeks
Gerbes et al., 2003	R, DB, PC	60	100	All	56.1	76.8	127.3	Lixivaptan 100 or 200 mg/d	Placebo	1	1
Wong et al., 2003	R, SB, PC	33	100	All	52.7	71.5	124.5	Lixivaptan 50, 25, or 500 mg/d	Placebo	1	1
Gines et al., 2008a	R, SB, PC	139	100	All	NA	NA	NA	Satavapan 5–50 mg/d	Placebo	52	52
Gines et al., 2008b	R, DB, PC	110	100	All	57.1	70.5	126.4	Satavapan 5, 12.5, or 25 mg/d	Placebo	2	2
Wong et al., 2009	R, SB, PC	234	100	All	NA	NA	134.5	Satavapan 5–50 mg/d	Placebo	52	52
Gines et al., 2010	R, DB, PC	148	100	None	57.3	67.2	136.6	Satavapan 5, 12.5, or 25 mg/d	Placebo	2	2
Wong et al., 2010	R, DB, PC	151	100	Partial (42.4%)	59.9	75.1	135.1	Satavapan 5, 12.5, or 25 mg/d	Placebo	12	12
Wong et al., 2012	R, DB, PC	463	100	Partial	56.5	70.6	137.0	Satavapan 5 or 10 mg/d	Placebo	52	52
Wong et al., 2012	R, DB, PC	497	100	Partial	58.3	70.6	136.0	Satavapan 5 or 10 mg/d	Placebo	52	52
Wong et al., 2012	R, DB, PC	240	100	Partial	56.3	65.8	135.0	Satavapan 5 or 10 mg/d	Placebo	52	52
Cárdenas et al., 2012	R, DB, PC	120	NA	All	53.5	73.3	128.7	Tolvaptan 15, 30, or 60 mg/d	Placebo	4	4
Sakaida et al., 2014	R, DB, PC	162	100	NA	68.5	62.4	135.5	Tolvaptan 7.5 mg/d	Placebo	1	2
Okita et al., 2014	R, DB, PC	101	100	NA	64.2	77.1	135.6	Tolvaptan 7.5, 15, or 30 mg/d	Placebo	1	2
Rai et al., 2017	R, OL	48	100	Partial	43.7	88.1	131.5	Tolvaptan 7.5 or 15 mg/d	Standard medical therapy	12	12
Uojima et al., 2017	R, OL	56	100	NA	69.3	56.8	135.0	Tolvaptan 7.5 mg/d	Standard medical therapy	1	4
Wang et al., 2018a	R, DB, PC	230	NA	Partial (42.6)	53.1	80.4	135.4	Tolvaptan 7.5, 15, or 30 mg/d	Placebo	1	26
Wang et al., 2018b	R, DB, PC	181	100	NA	51.1	75.8	NA	Tolvaptan 15 or 30 mg/d	Placebo	1	2
Wang et al., 2019a	R, OL	86	NA	All	47.5	64.0	128.7	Tolvaptan 15 mg/d	Standard medical therapy	4	8

**Table 2:** Study quality evaluation *via* Cochrane’s risk of bias tool.

	Random sequence generation	Allocation concealment	Blinding in performance	Blinding in outcome detection	Incomplete outcome data	Reporting bias	Other bias
Gerbes et al., 2003	Low	Unclear	Low	Low	Low	Low	Low
Wong et al., 2003	Unclear	Unclear	Low	High	Low	Low	High
Gines et al., 2008a	Unclear	Unclear	Low	High	Low	Low	Low
Gines et al., 2008b	Low	Unclear	Low	Low	Low	Low	Low
Wong et al., 2009	Unclear	Unclear	Low	High	Low	Low	Low
Gines et al., 2010	Low	Unclear	Low	Low	Low	Low	Low
Wong et al., 2010	Low	Low	Low	Low	Low	Low	Low
Wong et al., 2012	Unclear	Unclear	Low	Low	Low	Low	Low
Wong et al., 2012	Unclear	Unclear	Low	Low	Low	Low	Low
Wong et al., 2012	Unclear	Unclear	Low	Low	Low	Low	Low
Cárdenas et al., 2012	Unclear	Unclear	Low	Low	Low	Low	Low
Sakaida et al., 2014	Unclear	Unclear	Low	Low	Low	Low	Low
Okita et al., 2014	Unclear	Unclear	Low	Low	Low	Low	Low
Rai et al., 2017	Low	Low	High	High	Low	Low	Low
Uojima et al., 2017	Low	Low	High	High	Low	Low	Low
Wang et al., 2018a	Unclear	Unclear	Low	Low	Low	Low	Low
Wang et al., 2018b	Unclear	Unclear	Low	Low	Low	Low	Low
Wang et al., 2019a	Unclear	Unclear	High	High	Low	Low	Low

### Primary Outcome

**Figure 2:**
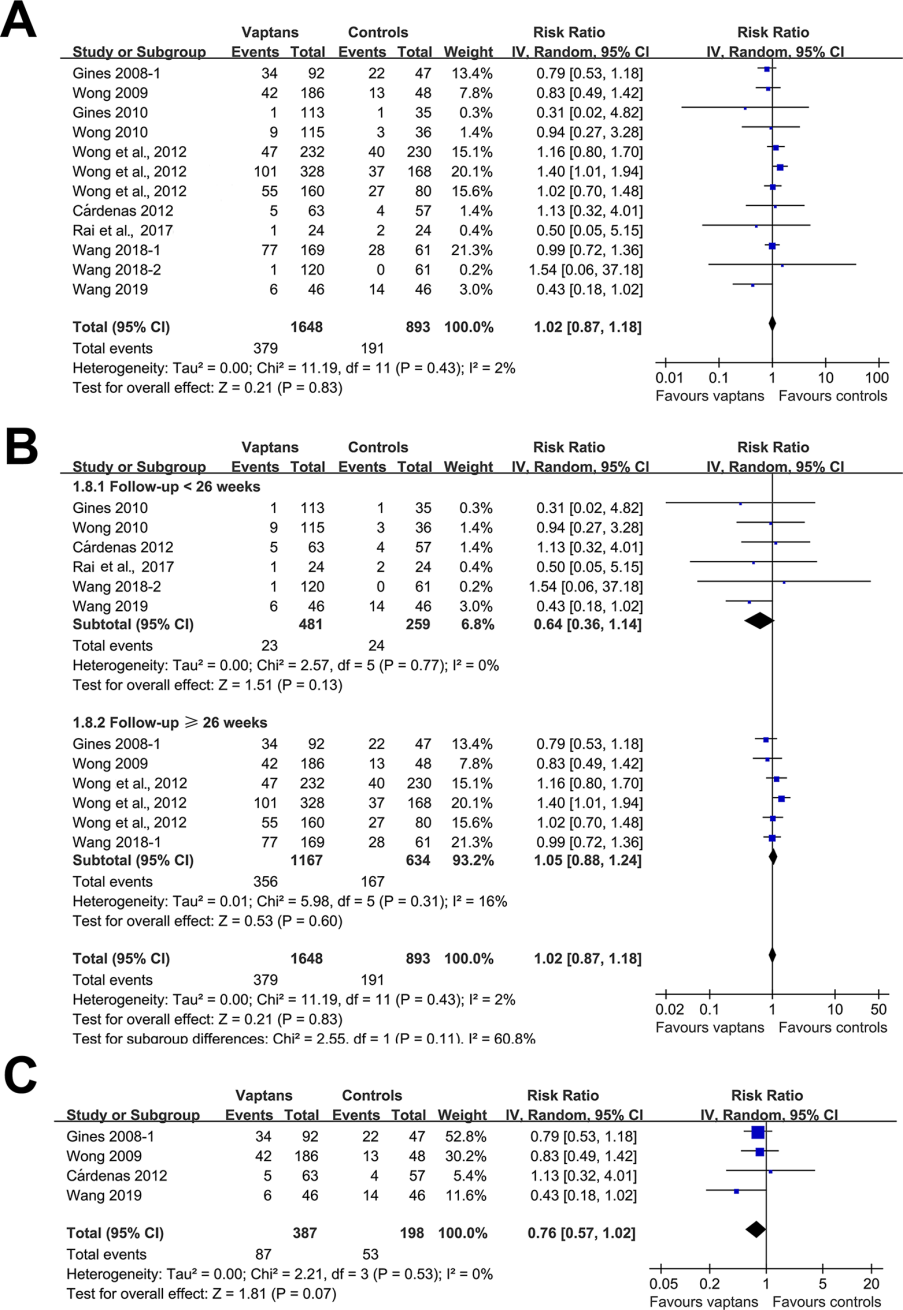
Forest plots for meta-analysis of the influence of vaptans on all-cause mortality in cirrhosis patients. **(A)** Overall meta-analysis. **(B)** Subgroup analysis according to the follow-up duration. **(C)** Sensitivity analyses limited to studies involving cirrhosis patients with hyponatremia before treatment.

A meta-analysis of 12 RCTs (Gines et al., 2008a; Wong et al., 2009; Gines et al., 2010; Wong et al., 2010; Cardenas et al., 2012; Wong et al., 2012; Rai et al., 2017; Wang et al., 2018a; Wang et al., 2018b; Wang et al., 2019a) including 2,541 patients showed that treatment with vaptans did not significantly affect the risk of all-cause mortality in cirrhosis patients (RR: 1.02, 95% CI: 0.87 to 1.08, p = 0.83; **Figure 2A**) without significant heterogeneity (P for Cochrane’s Q test = 0.83, I^2^ = 2%). Subgroup analyses showed that vaptans did not significantly affect mortality risk in cirrhosis patients in studies with a follow-up duration < 26 weeks (RR: 0.64, 95% CI: 0.36 to 1.14, p = 0.13; **Figure 2B**) or those with a follow-up duration ≥ 26 weeks (RR: 1.05, 95% CI: 0.88 to 1.24, p = 0.60; **Figure 2B**). However, meta-analysis restricted to studies of cirrhosis patients with hyponatremia before treatment (Gines et al., 2008a; Wong et al., 2009; Cardenas et al., 2012; Wang et al., 2019a) showed a trend of reduced mortality risk after vaptans treatment compared with the controls (RR: 0.76, 95% CI: 0.57 to 1.02, p = 0.07; **Figure 2C**).

### Secondary Outcomes

**Figure 3:**
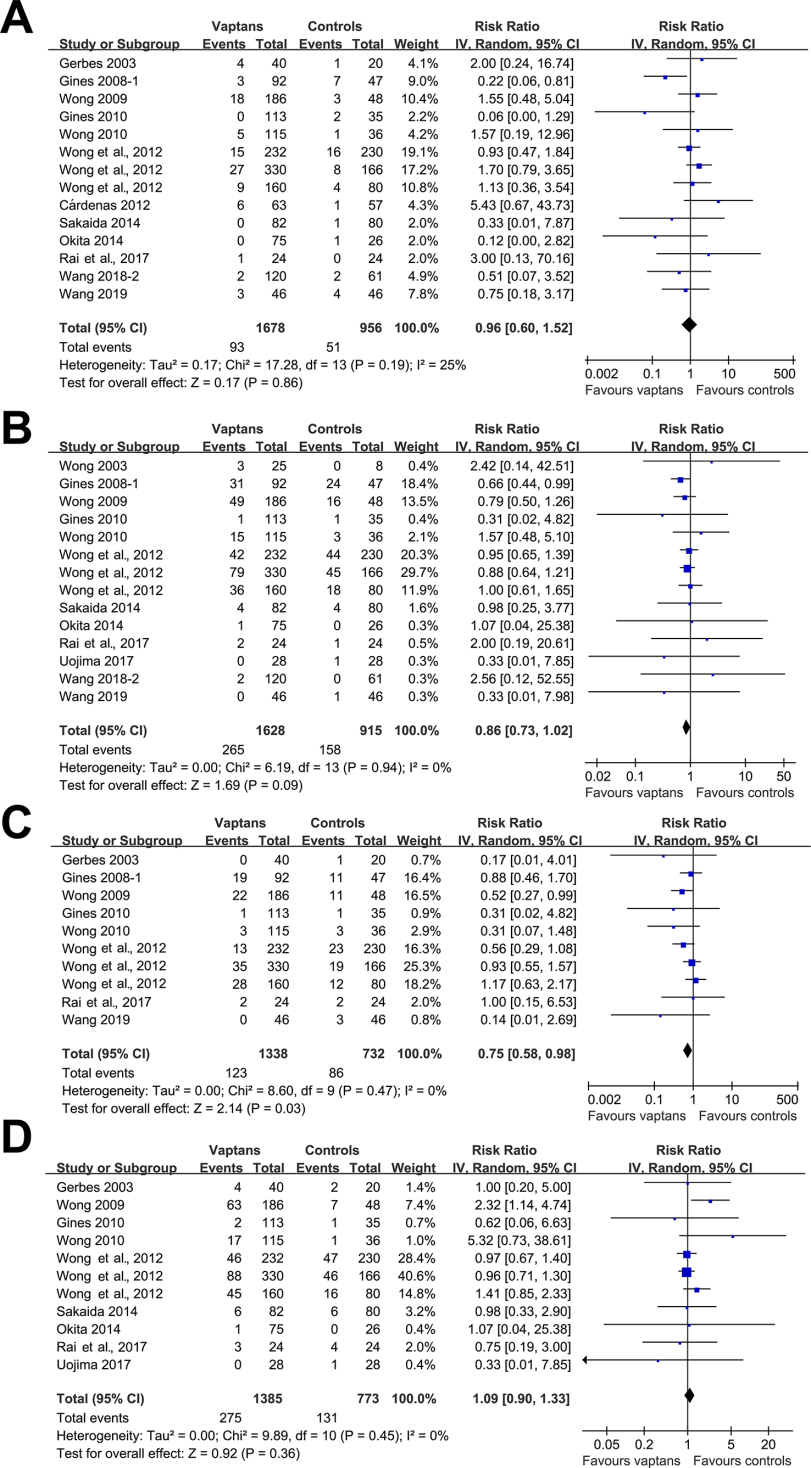
Forest plots for meta-analysis of the influence of vaptans on the risk of ascites-related complications in cirrhosis patients. **(A)** Incidence of variceal bleeding. **(B)** Incidence of hepatic encephalopathy. **(C)** Incidence of spontaneous bacterial peritonitis. **(D)** Incidence of hepatorenal syndrome or renal failure.

Meta-analyses with 10 to 14 studies showed that treatment with vaptans did not affect the incidence of VB (RR: 0.96, 95% CI: 0.60 to 1.52, p = 0.86; I^2^ = 25%; **Figure 3A**), reduced the incidence of HE (RR: 0.86, 95% CI: 0.73 to 1.02, p = 0.09; I^2^ = 0%; **Figure 3B**), significantly reduced the incidence of SBP (RR: 0.75, 95% CI: 0.58 to 0.98, p = 0.03; I^2^ = 0%; **Figure 3C**), but did not significantly affect the risk of RF (RR: 1.09, 95% CI: 0.90 to 1.33, p = 0.36; I^2^ = 0%; **Figure 3D**) in cirrhosis patients. No significant heterogeneity was detected among the included studies.

### Safety Outcomes

**Figure 4:**
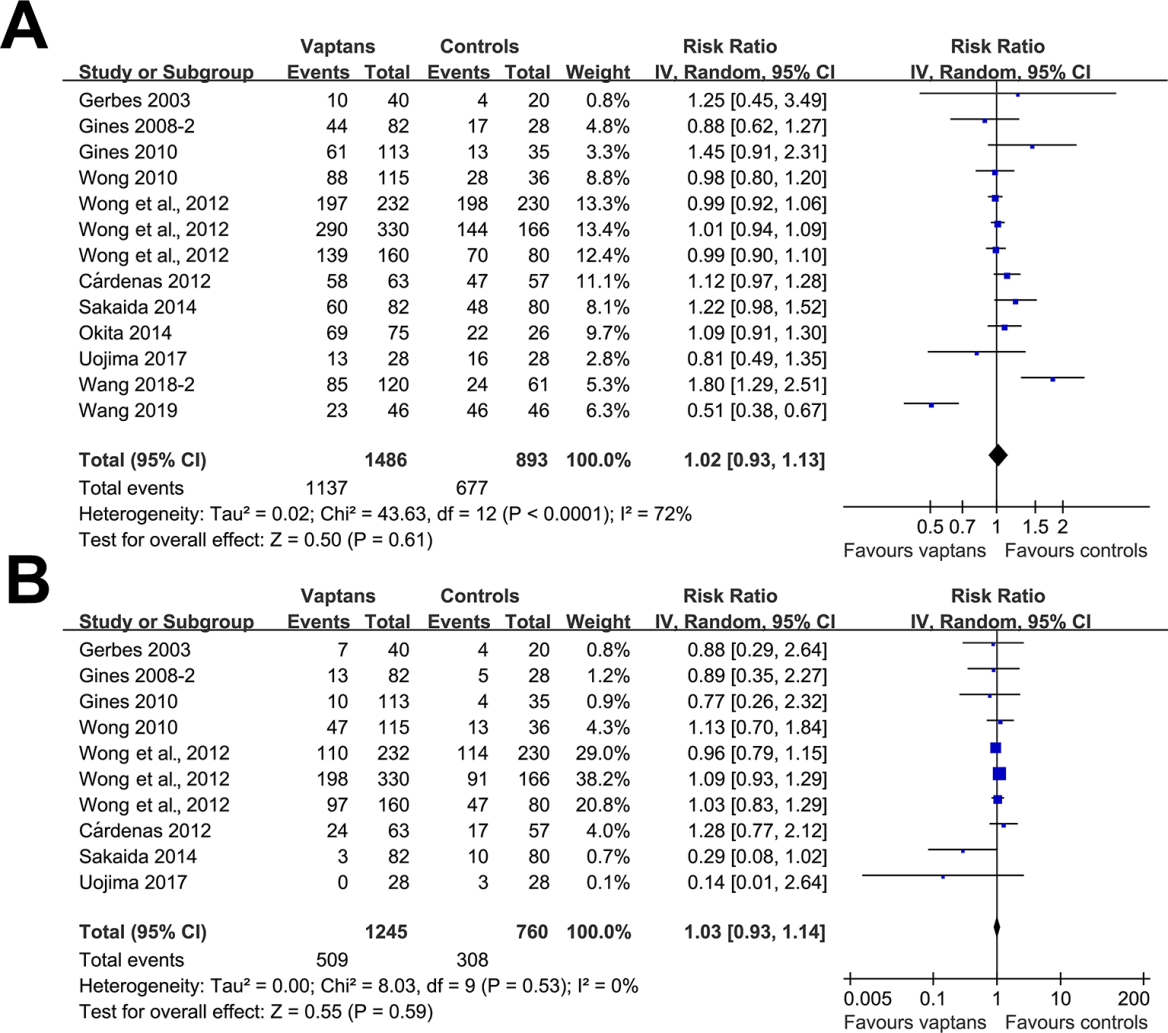
Forest plots for meta-analysis of the influence of vaptans on the risk of adverse events in cirrhosis patients. **(A)** Incidence of total adverse events. **(B)** Incidence of serious adverse events.

Pooled results showed that treatment with vaptans did not significantly affect the incidence of any adverse events (RR: 1.02, 95% CI: 0.93 to 1.13, p = 0.61; I^2^ = 72%; **Figure 4A**) or severe adverse events (RR: 1.03, 95% CI: 0.93 to 1.14, p = 0.59; I^2^ = 0%; **Figure 4B**).

### Publication Bias

**Figure 5:**
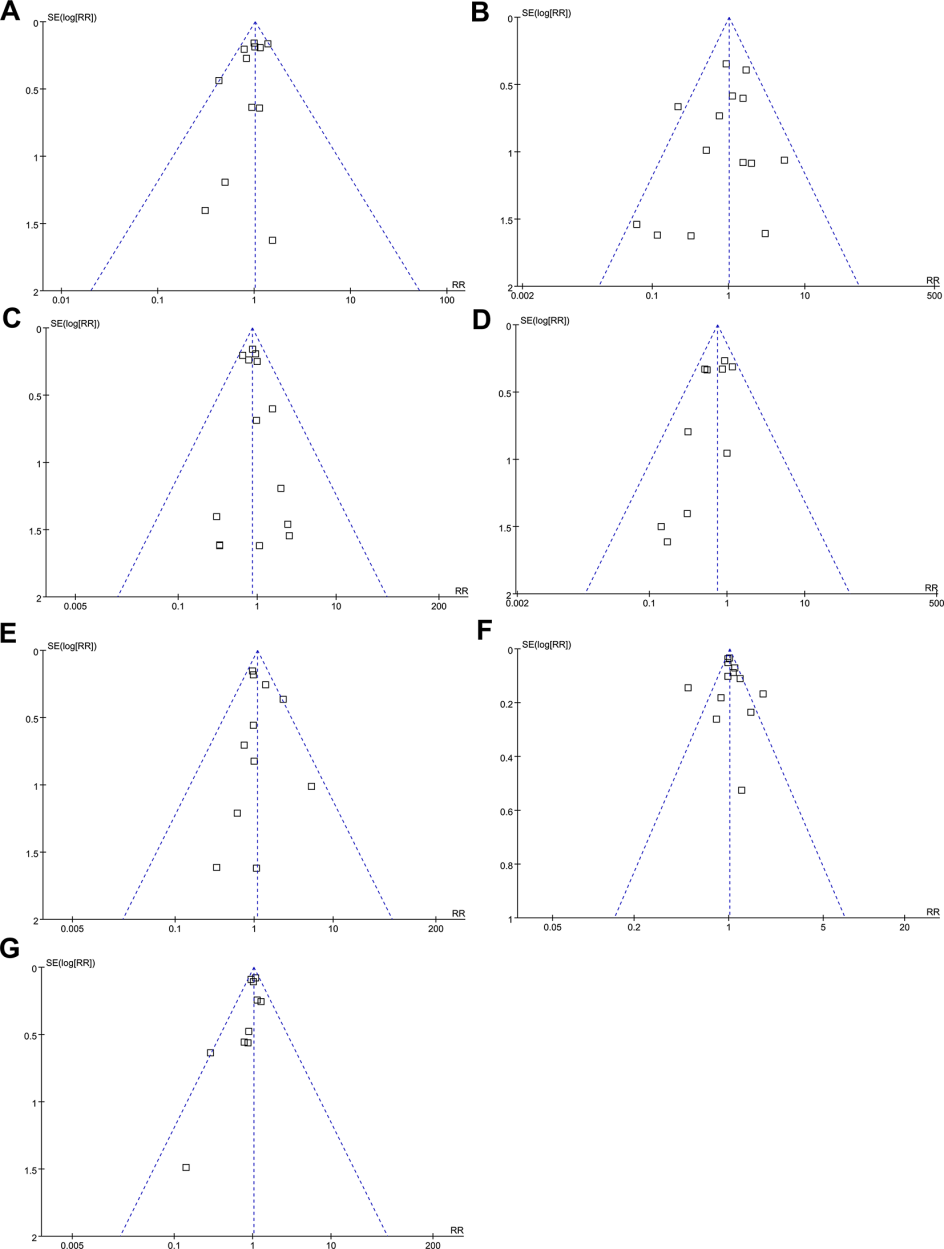
Funnel plots for meta-analyses of the influence of vaptans on the clinical outcomes in cirrhosis patients. **(A)** Incidence of all-cause mortality. **(B)** Incidence of variceal bleeding. **(C)** Incidence of hepatic encephalopathy. **(D)** Incidence of spontaneous bacterial peritonitis. (E) Incidence of hepatorenal syndrome or renal failure. **(F)** Incidence of total adverse events. **(G)** Incidence of serious adverse events.

Funnel plots for the meta-analysis regarding the influence of vaptans on the outcomes of all-cause mortality, incidence of VB, HE, SBP, and RF, and risk of any adverse events and severe adverse events are shown in **Figures 5A–G**. The plots are symmetric on visual inspection, indicating no significant publication biases. These findings were further confirmed by the results of Egger’s regression tests (p for publication bias = 0.219 ∼ 0.658).

## Discussion

In this study, by including all available RCTs, we found that treatment with vaptans is not associated with improved survival in cirrhosis patients, and the results were consistent with studies with short-term and long-term follow-up durations. Moreover, sensitivity analyses limited to studies including cirrhosis patients with hyponatremia before treatment showed a trend of reduced all-cause mortality risk in cirrhosis patients allocated to the vaptans group compared to those allocated to the control group (RR = 0.76, p = 0.07). Regarding the impact of vaptans on the complications related to ascites, we found that treatment with vaptans may reduce the risk of HE and SBP, but did not significantly affect the incidence of VB or RF. As for the safety outcomes, treatment of cirrhosis patients with vaptans did not significantly affect the risks of any adverse events or serious adverse events. Taken together, our findings suggested that treatment with vaptans is not associated with improved survival in cirrhosis patients, although it may reduce the risk of HE and SBP in these patients.

The beneficial effects of vaptans on ascites and hyponatremia have been confirmed in two previous meta-analyses. Dahl *et al*. performed a meta-analysis of 12 RCTs in 2012, and showed that treatment with vaptans increased serum sodium, reduced body weight, and reduced the time to first paracentesis (Dahl et al., 2012). Another meta-analysis including 14 RCTs in 2015 confirmed these results by showing that vaptans also significantly reduced abdominal girth and improved the proportions of patients with corrected hyponatremia after treatment (Yan et al., 2015). However, none of the above meta-analyses showed a significant effect on the mortality risk after treatment with vaptans. Our study, which additionally included recently published RCTs, also failed to demonstrate improved survival in cirrhosis patients after treatment with vaptans. Moreover, subsequent analyses showed consistent results in short-term and long-term follow-up studies. Interestingly, an exploratory analysis showed a trend of improved survival after vaptan treatment for cirrhosis patients with hyponatremia at baseline. Some explanations for the above findings may be considered. Firstly, although we included 18 RCTs that contained over 3,000 cirrhosis patients, since most of the studies had limited sample sizes and were not designed with a primary outcome of mortality, this meta-analysis remains statistically underpowered to detect a significant impact from vaptans on clinical outcomes. Secondly, the studies were heterogeneous regarding the characteristics of the cirrhosis patients, particularly the baseline sodium levels. Previous studies showed that the improvement or correction of hyponatremia is associated with improved survival in patients with hyponatremia caused by various diseases, including acute decompensated heart failure (Hauptman et al., 2013; Wang et al., 2019b) and cirrhosis (Corona et al., 2015). A recent cohort study showed that an increased serum sodium level after 1-month tolvaptan treatment may positively influence the mortality risk in cirrhosis patients with hyponatremia (Hayashi et al., 2018). Therefore, although current evidence does not support the beneficial effects of vaptans on survival in cirrhosis patients, vaptans may reduce the mortality risk in cirrhosis patients with hyponatremia at baseline and correct hyponatremia after treatment. Future RCTs with large sample sizes are needed to validate these hypotheses. Another important finding from the current analyses is that vaptans did not significantly affect the risks of adverse events compared to placebo or standard medical treatment, indicating the safety of vaptans. These results suggested that the use of vaptans should be considered for cirrhosis patients with ascites or hyponatremia.

We found that vaptans may reduce the risk of HE and SBP in patients with cirrhosis, which was not observed in previous meta-analysis (Dahl et al., 2012). Since both the pathogenesis of HE and SBP are related to the severity of ascites, it could be estimated that vaptans reduces the risk of HE and SBP by alleviating ascites. Moreover, it has been indicated in recent studies that hyponatremia correction by tolvaptan in cirrhosis patients is associated with the improvements of cognitive, quality of life, brain edema in MRI, and companion burden (Ahluwalia et al., 2015; Cardenas and Riggio, 2015), suggesting that the correction of hyponatremia may also be the underlying mechanism for the beneficial effects of vaptans on HE risk. This is further supported by a recent study which showed that the improvement of hyponatremia in patients with cirrhosis leads to an increase in the speed of complex information processing (Watson et al., 2019). Whether other mechanisms are involved in the benefits of vaptans on HE and SBP deserves to be investigated in future studies.

Results of meta-analysis showed that using of vaptans did not seem to increase the risk of adverse events in cirrhosis patients. However, some concerning has been raised by the U.S. Food and Drug Administration, which should be considered for the clinicians. For example, for initiating and reinitiating vaptans therapy, hospitalization is required to monitor serum sodium and volumes. Moreover, rapid correction of hyponatremia should be avoided, because it may osmotic demyelination resulting in dysarthria, mutism, dysphagia, lethargy, affective changes, spastic quadriparesis, seizures, coma, and even death. While for patients with cirrhosis, moderate and severe hepatic impairment do not affect exposure to tolvaptan to a clinically relevant extent, and no dose adjustment of tolvaptan is necessary.

Our study has limitations which should be considered when interpreting the results. Firstly, as mentioned before, most of the studies included have a limited scale and were not designed with a primary outcome of mortality. The effects of vaptans on survival in cirrhosis patients should be evaluated in large-scale RCTs with adequate statistical power. Secondly, whether the baseline characteristics of cirrhosis patients modify the impact of vaptans on survival should be investigated, particularly for those with hyponatremia before treatment who respond well to vaptans. Finally, the optimal doses and durations for vaptans in these patients should also be determined for clinical practice. To determine the effect of baseline characteristics of responders and optimal dose of vaptans, an individual patient data based meta-analysis should be performed.

In conclusion, the results of our meta-analysis showed that treatment with vaptans is not associated with improved survival in cirrhosis patients, although it may reduce the risk of HE and SBP in these patients. Moreover, vaptans are safe for cirrhosis patients and should be considered for cirrhosis patients with ascites or hyponatremia before treatment.

## Data Availability Statement

The raw data supporting the conclusions of this manuscript will be made available by the authors, without undue reservation, to any qualified researcher.

## Author Contributions

ML and ZB carried out the acquisition and analysis of data, and drafting the manuscript. ZH participated in the design and helped to revise the manuscript. All authors have read and approved the final version of the manuscript prior to submission.

## Conflict of Interest

The authors declare that the research was conducted in the absence of any commercial or financial relationships that could be construed as a potential conflict of interest.
